# Quality of Life Assessment Among Patients Living With Hepatosplenic Schistosomiasis and Schistosomal Myeloradiculopathy

**DOI:** 10.3389/fmed.2021.629484

**Published:** 2021-06-01

**Authors:** Silvana Júnia Roriz, Thiago Almeida Pereira, Guilherme Vaz de Melo Trindade, Júlia Fonseca de Morais Caporali, José Roberto Lambertucci

**Affiliations:** ^1^Graduate Program in Infectious Diseases and Tropical Medicine, School of Medicine, Federal University of Minas Gerais, Belo Horizonte, Brazil; ^2^Institute for Stem Cell Biology and Regenerative Medicine, Stanford University School of Medicine, Stanford, CA, United States; ^3^Physiology, School of Medicine, National University of Ireland, Galway, Galway, Ireland; ^4^Department of Internal Medicine, School of Medicine, Federal University of Minas Gerais, Belo Horizonte, Brazil

**Keywords:** hepatosplenic schistosomiasis, schistosomal myeloradiculopathy, schistosomiasis mansoni, quality of life, WHOQOL-BREF

## Abstract

Schistosomiasis is a major public health problem in tropical areas of the world. Health-related quality of life (HRQOL) measurement is being widely used to evaluate the impact of a disease or treatment in several aspects of daily life. However, few studies evaluated the impact of severe forms of schistosomiasis on HRQOL of affected individuals and compared them to healthy controls with a similar socio-demographic background. Our aims were to evaluate the HRQOL in patients with hepatosplenic schistosomiasis (HS) and schistosomal myeloradiculopathy (SMR) and healthy volunteers (HV) and determine if clinical complications of the disease are associated with HRQOL scores. We interviewed and evaluated the HRQOL in 49 patients with HS, 22 patients with SMR, and 26 HV from an outpatient clinic of the Federal University of Minas Gerais University Hospital using the WHOQOL-BREF questionnaire. SMR and HS patients had a significantly lower overall quality of life score when comparing with the HV control group (*p* = 0.003 and *p* = 0.005, respectively). Multivariate ordinal regression model adjusted for sex, age, and educational level indicated that HS and SMR patients have three and five times more chances of having a lower quality of life than healthy volunteers (Odds Ratio 3.13 and 5.04, respectively). There was no association between complications of HS disease and quality of life scores. In contrast, worse quality of life was observed in SMR patients that presented back or leg pain, leg paresthesia, and bladder dysfunction. In conclusion, HS and SMR significantly impact the overall quality of life of the affected individuals, reinforcing the importance of efforts to control and eradicate this debilitating disease and suggesting that multidisciplinary clinical management of schistosomiasis patients would be more appropriate and could potentially improve patient's quality of life.

## Introduction

Schistosomiasis continues to be a significant health threat in tropical areas of the world, affecting over 250 million people (1). In Brazil, the country harboring most cases in the Americas, it is estimated that 1.6–6 million people are infected with *Schistosoma mansoni*, and over 25 million individuals are at risk of infection (2–5).

Infected individuals have various clinical manifestations that generally cluster into three distinct forms of the disease: acute, hepatointestinal, and hepatosplenic schistosomiasis (6, 7). Ectopic forms of the disease can occur, and schistosomal myeloradiculopathy is the most severe and disabling ectopic manifestation of schistosomiasis (6–9).

The acute phase is rarely symptomatic in infected individuals from endemic areas due to early childhood infection and exposure to schistosome antigens and anti-schistosome antibodies in-utero and breast milk (6, 10). In contrast, individuals from non-endemic areas that become infected with *S. mansoni* usually develop a symptomatic acute form of schistosomiasis, characterized by a systemic hypersensitivity reaction against the migrating schistosomula and mature eggs (6, 10). The majority of chronically infected individuals develop hepatointestinal schistosomiasis (90–96%), a mild form of the disease consisted of granulomatous hepatic and intestinal inflammation, absent or discrete hepatosplenomegaly, and minimal liver periportal fibrosis without portal hypertension (7, 11). The severe form of the disease, named hepatosplenic schistosomiasis, is observed in a small percentage of infected individuals (4–10%) and is characterized by hepatosplenomegaly, severe liver periportal fibrosis, portal hypertension, and thrombocytopenia (7, 11). Schistosomal myeloradiculopathy is the most common manifestation of neuroschistosomiasis associated with *S. mansoni* infection (8, 9). This ectopic form of the disease can be observed in patients with acute or hepatointestinal schistosomiasis but is rarely observed in hepatosplenic patients (8, 9). Patients with schistosomal myeloradiculopathy frequently present lumbar and lower limb pain, paraparesis, paresthesia, and bladder dysfunction (urinary retention and other urological complications such as hydronephrosis, bladder calculi and recurrent urinary tract infections) (8, 9). The lesions are caused by a granulomatous inflammation induced by the eggs trapped in the central nervous system (8, 9).

Health-related quality of life (HRQOL) measurement is being widely used to evaluate the impact of a disease or treatment in several aspects of daily life (12, 13). Assessment of HRQOL, together with morbidity and mortality measurements, is crucial to determine the disease's burden in the community, evaluate interventions and standard health care policies, and identify areas that require further improvement (12–16). Generic HRQOL, such as WHOQOL-BREF or EQ-5D-3L, has been validated in different languages, cultural settings, and different diseases, including schistosomiasis (12–25).

Hepatosplenic schistosomiasis and schistosomal myeloradiculopathy are considered the most severe manifestations of *S. mansoni* infection (7–9). However, few studies evaluated the impact of severe forms of schistosomiasis on HRQOL of affected individuals and compared to healthy controls with a similar socio-demographic background (15, 17–19, 22, 26).

In Brazil, a recent study investigated the quality of life and quality-adjusted life years in chronic schistosomiasis patients and observed a similar reduction of HRQOL in hepatointestinal and hepatosplenic patients (22). Although some studies evaluate HRQOL in hepatointestinal patients with different intensity of infection, or hepatosplenic patients with different degrees of morbidity, no study evaluated schistosomal myeloradiculopathy's impact on HRQOL (15, 17–19, 22, 26). Therefore, our aims were to evaluate the HRQOL in healthy volunteers and patients with hepatosplenic schistosomiasis and schistosomal myeloradiculopathy and determine if the disease's clinical manifestations are associated with HRQOL scores.

## Materials and Methods

### Study Design and Ethical Considerations

This was a descriptive, observational cross-sectional study approved by the Federal University of Minas Gerais Research Ethics Committee (Protocol 692545/2014) and fully conducted according to the guidelines in the Declaration of Helsinki from the World Medical Association (27). All patients and healthy volunteers agreed to participate and provided informed consent to be included in this study. Interviews were conducted in a private room to ensure the patient's privacy.

### Study Population and Inclusion Criteria

We recruited patients with hepatosplenic schistosomiasis mansoni (HS) and with schistosomal Myeloradiculopathy (SMR) previously diagnosed and currently receiving medical care at Orestes Diniz Infectious and Parasitic Diseases Treatment Center, an outpatient clinic of the Federal University of Minas Gerais University Hospital (Belo Horizonte, Brazil). The caregivers accompanying patients to their clinic appointment served as non-infected controls and were included in the healthy volunteer group (HV). The sample size was calculated based on a pilot study conducted with 10 volunteers from each group (HS, SMR and HV) that indicated a minimum sample size 21 of participants for each study group, considering a 95% two-sided confidence level and 80% power. Adult individuals (18–65 years old) of both genders that fit the inclusion criteria for each group were invited to participate in the study. The following exclusion criteria were applied: individuals diagnosed with other infectious diseases, other liver diseases, other forms of myeloradiculopathy, and noticeable memory loss. HS group consisted of 49 adult patients, of both genders, with a previous hepatosplenic schistosomiasis diagnosis based on parasitological stool test (PST), rectal biopsy, liver biopsy serological tests and ultrasonography of the abdomen and upper digestive endoscopy. For the SMR group, 22 adult patients, of both genders, with a previous diagnosis of SMR based on PST, history of contact with *S. mansoni* contaminated freshwater, serological tests, examination of cerebrospinal fluid, clinical evidence of spinal cord injury confirmed by magnetic resonance of the lumbar spine and differential diagnosis to exclude other causes of myelitis (spinal cord trauma, tumor, vitamin B12 deficiency, antiphospholipid syndrome, diabetic or autoimmune vasculitis, HIV, HTLV, HCV, HSV, HBV, syphilis, tuberculosis, neurocysticercosis, medullary abscess, syringomyelia, herniated lumbar disc, polyradiculoneuritis, demyelinating diseases, radiotherapy) (8, 28), were included. The HV group consisted of 26 adults of both genders, that were the caregivers of patients undergoing treatment at the clinic and were apparently healthy, negative for *S. mansoni* infection, and without a previous diagnosis of schistosomiasis.

### Assessment of Health-Related Quality of Life

All participants were interviewed on the day of their routine appointment at the outpatient clinic and answered a clinical-socio-demographic questionnaire and the WHOQOL-BREF questionnaire, the shortened version of the World Health Organization Quality of Life questionnaire (WHOQOL-100) (12). The WHOQOL-BREF contains 26 questions, with two questions referring to the overall quality of life and the remaining questions related to physical, psychological, social and environmental domains (12). The Portuguese language version of WHOQOL-BREF has been validated and is widely used in Brazil as an appropriate tool to measure HRQOL in clinical settings (29–33).

### Statistical Analysis

The data collected was stored in a computerized database using Excel software (Microsoft Office 2013) and analyzed using the Statistical Package for Social Sciences software (SPSS), version 20.0 (SPSS, IBM Company, Chicago, IL). Significance was accepted at the 0.05 level. Frequency distribution tables were constructed, and central tendency and variability measures were calculated for the total sample and for each study group. Kolmogorov-Smirnov normality was used to determine if the variables have a Gaussian distribution. Categorical variables were compared using Pearson's chi-square or exact Fisher test, and the numerical variables using the Kruskal-Wallis test. For multiple comparisons among groups, Mann-Whitey test with Bonferroni correction was used. The proportional odds logistic regression model was used to estimate the odds ratio with 95% confidence intervals.

## Results

The socio-demographic data of the study population are depicted in Table 1. All groups were similar regarding relationship status, race/skin color, household income, and BMI. However, the study groups differed when evaluating the variables sex, age, educational levels, and occupation. Males were predominant in the hepatosplenic schistosomiasis and schistosomal myeloradiculopathy groups, while females were the majority among the healthy volunteers. Although similar age distribution was observed in SMR and healthy volunteers, HS patients were, on average, about 7 years older. Healthy volunteers presented higher education levels (secondary and tertiary) than schistosomiasis patients. However, a higher frequency of college level educated individuals was observed in the SMR group. A higher frequency of individuals currently on sick leave were observed in the SMR group, and in SMR and HS groups, there were more retired and unemployed individuals than the HV group.

**Table 1:** Socio-demographic data of schistosomiasis patients and healthy volunteers included in this study.

	**Hepatosplenic** **Schistosomiasis** **(*****n*** **= 49)**		**Schistosomal** **Myeloradiculopathy** **(*****n*** **= 22)**		**Healthy** **Volunteers** **(*****n*** **= 26)**		**Total** **(*****n*** **= 97)**		
	***n***	**%**	***n***	**%**	***n***	**%**	***n***	**%**	***p*-value**
**Sex**									
Male	29	59.2	19	86.4	7	26.9	55	56.7	<0.001^*^
Female	20	40.8	3	13.6	19	73.1	42	43.3	
**Relationship status**									
With partner	30	61.2	13	59.1	16	61.5	59	60.8	1.000^*^
Without partner	19	38.8	9	40.9	10	38.5	38	39.2	
**Race/skin color**									
White-skinned	30	61.2	15	68.2	19	73.1	64	66.0	0.570^*^
Black/brown-skinned	19	38.8	7	31.8	7	26.9	33	34.0	
**Educational level**									
Primary	36	73.5	14	63.6	10	38.5	60	61.9	0.019^**^
Secondary	11	22.4	5	22.7	14	53.8	30	30.9	
Tertiary	2	4.1	3	13.6	2	7.7	7	7.2	
**Occupation**									
Employed	26	53.1	7	31.8	21	80.8	54	55.7	0.002^**^
Unemployed	5	10.2	2	9.1	1	3.8	8	8.2	
Sick leave	6	12.2	9	40.9	0	0.0	15	15.5	
Retired/pensioner	12	24.5	4	18.2	4	15.4	20	20.6	
**Household income**									
1–3 minimum wage	44	91.7	18	90.0	25	96.2	87	92.6	0.772^**^
4–6 minimum wage	4	8.3	2	10.0	1	3.8	7	7.4	
**Age (years)**									
Average	48.9		41.3		41.8		45.3		0.023^***^
Standard deviation	10.7		13.3		13.7		12.6		
Median	51.0		44.5		41.5		47.0		
**BMI**									
Average	24.4		25.7		25.5		24.7		0.289^***^
Standard deviation	4.2		4.8		4.5		4.4		
Median	23.5		25.9		23.2		23.9		

* *Pearson's chi-squared test;*

** *Fisher's exact test;*

*** *Kruskal-Wallis test. The bold values are the p-values that are statistically significant*.

The WHOQOL-BREF questionnaire analysis demonstrated that schistosomiasis patients had worse perceived health-related quality of life when taking into account the overall quality of life score (Table 2, *p* = 0.007). There were no statistical differences among groups when the physical, environmental, social, and psychological domains of WHOQOL-BREF were evaluated. Multiple comparison analysis demonstrated that SMR or HS had a significantly lower overall quality of life score when comparing with the HV group (*p* = 0.003 and *p* = 0.005, respectively). Although SMR had a lower overall quality of life score when compared to HS, this difference was not statistically significant (*p* = 0.363). Further analysis using the multivariate ordinal regression model and adjusting for sex, age, and educational level indicate that HS and SMR patients have three and five times more chances of having a lower quality of life than healthy volunteers (Table 3; Odds Ratio 3.13 and 5.04, respectively). Our model showed good agreement according to the deviance and parallel lines test (Table 3).

**Table 2:** Analysis of perceived quality of life in schistosomiasis patients and non-infected volunteers based on the short form of the World Health Organization Quality of Life questionnaire (WHOQOL-BREF).

**WHOQOL-BREF**	**Hepatosplenic**	**Schistosomal**	**Healthy**	
**Domains**	**Schistoso- miasis**	**Myeloradi-culopathy**	**Volunteers**	
	**(*n* = 49)**	**(*n* = 22)**	**(*n* = 26)**	*p*-value^*^
**Physical**				
25th percentile	50.0	45.5	53.6	0.251
Median score	53.6	51.8	62.5	
75th percentile	71.4	67.9	71.4	
**Psychological**				
25th percentile	66.7	57.3	61.5	0.852
Median score	70.8	70.8	72.9	
75th percentile	79.2	83.3	75.0	
**Social**				
25th percentile	66.7	58.3	58.3	0.714
Median score	75.0	75.0	83.3	
75th percentile	91.7	93.8	100.0	
**Environmental**				
25th percentile	59.4	52.3	50.0	0.326
Median score	68.8	73.4	64.1	
75th percentile	79.7	85.2	78.9	
**Overall quality of life**				
25th percentile	50.0	37.5	71.9	**0.007**
Median score	62.5	50.0	75.0	
75th percentile	75.0	75.0	75.0	

* *Kruskall-Wallis test. The bold values are the p-values that are statistically significant*.

**Table 3:** Multivariate ordinal regression model for overall quality of life based on the short form of the World Health Organization Quality of Life questionnaire (WHOQOL-BREF) in patients with schistosomiasis compared to non-infected volunteers.

**Patient Group**	**Odds ratio**	**95% CI**	*p*-value^*^
Hepatosplenic schistosomiasis	3.13	1.05–9.29	**0.040**
Schistosomal myeloradiculopathy	5.04	1.34–18.89	**0.017**

* *Multivariate ordinal regression model adjusted for sex, age and educational level.*

*Deviance test = 0.678; Paralel line test = 0.265. The bold values are the p-values that are statistically significant.*

Finally we examined if complications of HS or SMR were associated with worse quality of life scores. For HS patients, there was no association between complications of the disease and quality of life scores (Table 4). In contrast, worse quality of life was observed in SMR patients that presented with back or leg pain, leg paresthesia, and bladder dysfunction (Table 5).

**Table 4:** Analysis of overall quality of life score and clinical complications of hepatosplenic schistosomiasis.

	**Overall quality of life score (WHOQOL-BREF)**						
	**<50**		**>50 <75**		**>75**		
	**(n = 20)**		**(n = 26)**		**(n = 3)**		p-value^*^
	**n**	**%**	**n**	**%**	**n**	**%**	
**Gastrointestinal bleeding**							
No	9	45	11	42.3	1	33.3	0.999
Yes	11	55	15	57.7	2	66.7	
**Other bleeding**							
No	9	45	13	50	1	33.3	0.903
Yes	11	55	13	50	2	66.7	
**Required blood transfusion**							
No	10	50	15	57.7	2	66.7	0.902
Yes	10	50	11	42.3	1	33.3	
**Required sclerotherapy**							
No	11	55	10	38.5	2	66.7	0.465
Yes	9	45	16	61.5	1	33.3	
**Portal Hypertension**							
No	4	20	7	26.9	2	66.7	0.240
Yes	16	80	19	73.1	1	33.3	
**Splenectomy**							
No	16	80	18	69.2	2	66.7	0.592
Yes	4	20	8	30.8	1	33.3	
**Thrombocytopenia**							
No	1	5	4	15.4	0	0	0.597
Yes	19	95	22	84.6	3	100	
**Ascites**							
No	16	80	24	92.3	3	100	0.585
Yes	4	20	2	7.7	0	0	
**Peripheral edema**							
No	17	85	22	84.6	3	100	0.999
Yes	3	15	4	15.4	0	0	
**Collateral circulation**							
No	14	70	19	73	2	66.7	0.884
Yes	6	30	6	23	1	33.3	
**Liver**							
Palpable	10	50	14	53.8	2	66.7	0.999
Not Palpable	10	50	12	46.2	1	33.3	
**Spleen**							
Palpable	6	30	11	42.3	1	33.3	0.717
Not Palpable	14	70	14	53.8	2	66.7	
**Ultrasound examination**							
Normal	1	5	1	3.8	0	0	0.999
Liver fibrosis and splenomegaly	16	80	22	84.6	2	66.7	

* *Fisher's exact test*.

**Table 5:** Analysis of overall quality of life score and clinical complications of schistosomal myeloradiculopathy.

	**Overall quality of life score (WHOQOL-BREF)**						
	**<50**		**>50 <75**		**>75**		
	**(*****n*** **= 12)**		**(*****n*** **= 7)**		**(*****n*** **= 3)**		*p*-value^*^
	***n***	**%**	***n***	**%**	***n***	**%**	
**Back pain**							
Absent	1	8.3	2	28.6	3	100.0	**0.013**
Present	11	91.7	5	71.4	0	0.0	
**Leg pain**							
Absent	3	25.0	1	14.3	3	100.0	**0.048**
Present	9	75.0	6	85.7	0	0.0	
**Leg paralysis**							
Absent	11	91.7	6	100.0	3	100.0	1
Present	1	8.3	0	0.0	0	0.0	
**Leg paresthesia**							
Absent	2	16.7	1	14.3	3	100.0	**0.019**
Present	10	83.3	6	85.7	0	0.0	
**Bladder dysfunction**							
Absent	1	8.3	2	28.6	3	100.0	**0.013**
Present	11	91.7	5	71.4	0	0.0	
**Intestinal dysfunction**							
Absent	3	25.0	3	42.9	2	66.7	0.497
Present	9	75.0	4	57.1	1	33.3	
**Erectile dysfunction**							
Absent	3	33.3	1	14.3	3	100.0	0.06
Present	6	66.7	6	85.7	0	0.0	

* *Fisher's exact test. The bold values are the *p*-values that are statistically significant*.

## Discussion

Assessment of HRQOL in schistosomiasis patients is commonly used to evaluate therapeutic interventions and mass drug administration strategies in endemic areas (16, 19, 21, 23–25). However, studies that determine the burden of severe forms of schistosomiasis on patients' quality of life are scarce and rarely include an appropriate control group with similar socio-demographic background for comparison (15, 17, 18, 22, 26).

In the present study, we evaluated the HRQOL in healthy volunteers and patients with hepatosplenic schistosomiasis and schistosomal myeloradiculopathy. Although there was no significant difference among the groups on the scores in the four domains of the WHOQOL-BREF questionnaire, patients with schistosomiasis had a significantly lower overall quality of life score than healthy volunteers. SMR patients had lower scores of overall quality of life than HS, but this difference was not statistically significant. In addition, patients with HS had three times more chances to have lower scores of overall quality of life, while patients with SMR had five times more chances to present worse quality of life than healthy adults with similar socio-demographic backgrounds. To the best of our knowledge, our study is the first that addressed HRQOL in patients with SMR and compared the scores with HS patients and non-infected individuals.

Kamel et al. (26) evaluated *S. mansoni* and *S. haematobium* infection's impact on the quality of life and productivity in workers of a textile factory in Egypt. The authors used the WHOQOL-BREF questionnaire and demonstrated that infected individuals had lower scores in the physical, social, and environmental domains than non-infected workers (26). Furthermore, they observed a significant correlation between the severity of schistosomiasis and lower quality of life and productivity scores (26).

Furst et al. (18) investigated the self-reported quality of life using the WHOQOL-BREF questionnaire in adults with schistosomiasis, soil-transmitted helminthiasis, or non-infected volunteers in Côte d'Ivoire. Although the sample size of patients with schistosomiasis was small (187 participants, 2.1% infected with *S. mansoni* and 2.1% with *S. haematobium*), they observed that infection with *S. mansoni* reduced the overall quality of life of individuals by 16 points when compared to non-infected individuals, thus reinforcing that schistosomiasis have a significant impact on the quality of life of individuals (18). In our patient cohort, we observed that the median of the scores for overall quality of life in patients with HS and SMR patients were, respectively, 12.5 and 25 points lower than the scores for healthy volunteers.

In China, studies that used the EQ-5D plus questionnaire indicated that chronically infected patients with S. japonicum also had low quality of life scores and heavy disability weights that are associated with age, impaired work capacity, depression, anxiety, ascites, and active hepatitis B infection (15, 17). In our study, we did not find an association of lower quality of life scores and HS complications, but in SMR patients, lower scores were significantly associated with the presence of back or leg pain, leg paresthesia, and bladder dysfunction.

In Brazil, Barbosa and Pereira da Costa (34, 35) evaluated the impact of different forms of chronic schistosomiasis in the productivity of sugarcane cane cutters. Both studies were conducted prior to the standardization of HRQOL generic questionnaires and evaluated only the disease impact on the physical domain, and measured the productivity based on the salary received and tons of sugarcane collected by individuals from each group in one harvest (34, 35). The authors demonstrated that workers with hepatosplenic schistosomiasis had significantly lower productivity than workers with hepatointestinal form of the disease, indicating that hepatosplenic schistosomiasis negatively impacted the ability to work for those individuals (34, 35). We observed that HS patients had lower scores in the physical, social, and environmental domains than healthy volunteers, but this difference was not statistically different.

Nascimento et al. (22) conducted the first study of HRQOL in schistosomiasis patients in Brazil. HRQOL was investigated using the EQ-5D-3L questionnaire in 147 patients (56 hepatointestinal and 91 hepatosplenic). Although the authors did not include a non-infected control group, the study demonstrated that schistosomiasis is associated with lower scores in the pain/discomfort and anxiety/depression dimensions (22). Female patients and the presence of comorbidities were associated with worse quality of life, while there was no significant difference in the quality of life scores between hepatointestinal and hepatosplenic patients (22). In our study, none of the socio-demographic variables, including gender and the presence of comorbidities, were associated with poor quality of life. Only the variable “*S. mansoni* infection” was associated with lower scores of overall HRQOL.

This study indicated that HS and SMR patients had a higher chance of having a worse quality of life. However, our results should be taken with caution. The non-infected control group consisted of caregivers accompanying patients receiving medical care at our outpatient clinic. Although we observed significant differences among patients and non-infected controls, healthy volunteers also reported low scores of HRQOL. Cruz et al. (36) previously demonstrated that in Brazil lower quality of life is frequently observed in females, individuals from lower economic class, and individuals with lower education levels (36). The scores reported by Cruz et al. (36) for the physical, psychological, social and environmental domains of the WHOQOL-BREF questionnaire for healthy individuals from lower economic class (class D, household income = 2–4 minimum wage) were similar to our HV group (36). It has been reported that caregivers are subjected to psychological distress and have increased stress hormone levels and lower HRQOL than the general population (37). The HS, SMR, and healthy volunteer groups were fairly homogeneous regarding socio-demographic variables but differed in gender, age, educational levels, and occupation. We cautiously evaluated if the difference in the distribution of those variables influenced our results and adjusted the model to account for this caveat. Even though the groups were not as homogeneous as desirable, we were able to compare the groups and proceed with our analysis. Subsequent studies should avoid, if possible, discrepancies regarding the socio-demographic variables.

Some of the differences observed in the socio-demographic variables among the different groups are due to the inherent characteristics of the disease forms or related to the participants' professional occupation. HS patients are usually older than patients with other forms of schistosomiasis because severe liver fibrosis and portal hypertension take a long time to develop, and it usually requires multiple infections (7, 11). Symptomatic SMR is more frequently observed in males partly due to work-related exposure to *S. mansoni* in rural areas (8, 9). The HV group consisted of the caregivers accompanying patients to their visit to our outpatient clinic and were usually the patients' wives or daughters. SMR and HS are associated with high morbidity and are often incapacitating conditions; therefore, retirement, sick leave, and unemployment were more frequent among those groups. Unfortunately, we did not have access to patients with other clinical forms of schistosomiasis such as hepatointestinal schistosomiasis and symptomatic acute schistosomiasis. Future studies that include other forms of the disease and appropriately matched controls will be required to determine schistosomiasis' impact on HRQOL.

In conclusion, our results indicate that HS and SMR significantly impact the overall quality of life of the affected individuals, reinforcing the importance of efforts to control and eradicate this debilitating disease and suggesting that multidisciplinary clinical management of schistosomiasis patients would be more appropriate and could potentially improve patient's quality of life.

## Data Availability Statement

The raw data supporting the conclusions of this article will be made available by the authors, without undue reservation.

## Ethics Statement

The studies involving human participants were reviewed and approved by Federal University of Minas Gerais Research Ethics Committee. The patients/participants provided their written informed consent to participate in this study.

## Author Contributions

SR, TP, and JL designed the study, supervised patient recruitment, performed statistical analysis and wrote the manuscript. SR, GV, and JC recruited patients, performed the analytical tests, collected the data. All authors critically revised the manuscript and approved the final submission.

## Conflict of Interest

The authors declare that the research was conducted in the absence of any commercial or financial relationships that could be construed as a potential conflict of interest.
